# Evaluation of the Role of Dynamic Contrast-Enhanced MR Imaging for Patients with BI-RADS 3–4 Microcalcifications

**DOI:** 10.1371/journal.pone.0099669

**Published:** 2014-06-13

**Authors:** Yanni Jiang, Jianjuan Lou, Siqi Wang, Yi Zhao, Cong Wang, Dehang Wang

**Affiliations:** 1 Division of Radiology, The First Affiliated Hospital of Nanjing Medical University, Nanjing, China; 2 Division of Breast Surgery, The First Affiliated Hospital of Nanjing Medical University, Nanjing, China; 3 Division of Pathology, The First Affiliated Hospital of Nanjing Medical University, Nanjing, China; Stanford University School of Medicine, United States of America

## Abstract

**Objective:**

The purpose of study was to prospectively evaluate the diagnostic performance of dynamic contrast-enhanced MR imaging in the differentiation of malignant lesions from benign ones in patients with BI-RADS 3–4 microcalcifications detected by mammography.

**Materials and Methods:**

93 women with 100 microcalcifications had undergone breast MRI from June 2010 to July 2013. Subsequently, 91 received open biopsy and 2 received stereotactic vacuum-assisted biopsy. All results were compared with histological findings. The PPV, NPV and area under curve (AUC) of the mammography and breast MRI were calculated.

**Results:**

There were 31 (31.0%) BI-RADS 3 microcalcifications and 69 (69.0%) BI-RADS 4. The PPV and NPV of mammography is 65.2% (45/69) and 90.3% (28/31). The PPV and NPV of breast MRI was 90.2% (46/51) and 95.9% (47/49). Among 31 BI-RADS 3 microcalcifications, the PPV and NPV of breast MRI was 100% (3/3) and 100% (28/28). Among 69 BI-RADS 4 microcalcifications, the PPV and NPV of breast MRI was 89.6% (43/48) and 90.5% (19/21). The AUC of mammography and breast MRI assessment were 0.738 (95% CI, 0.639–0.837) and 0.931 (95% CI, 0.874–0.988) (*p*<0.05).

**Conclusion:**

Dynamic contrast-enhanced MR imaging of breast is able to be applied to predict the risk of malignance before follow-up for BI-RADS 3 microcalcifications and biopsy for BI-RADS 4 microcalcifications.

## Introduction

Microcalcifications represent one of the most important mammographic findings of breast carcinomas and are often the only mammographic signs of malignant breast lesion. According to the Breast Imaging Reporting and Data System (BI-RADS) [1], short-term mammographic follow-up is often recommended for probably benign microcalcifications (BI-RADS3). The positive predictive value (PPV) of BI-RADS 3 microcalcifications in mammography using stereotactic vacuum-assisted biopsy (SVAB) is only 2.7–7% [2]–[4]. Most patients with benign microcalcifications will worry about the lesions during the follow-up and some with malignant microcalcifications will be lost during the follow-up. In clinical practice, histological evaluation is required for suspiciously abnormal microcalcifications(BI-RADS 4). But the PPV of BI-RADS 4 microcalcifications is only 20–25% proved by biopsy [2], [5]–[6]. So it's better to decrease the rate of defaulters and to avoid unnecessary biopsy by some new noninvasive breast examination.

Compared with mammography, dynamic contrast-enhanced Magnetic Resonance Imaging (DCE MRI) is more sensitive to detect breast carcinomas in dense and heterogeneously dense breasts [7]. DCE MRI has been demonstrated to be with the high sensitivity of invasive ductal carcinoma approached to 100% [8]. However, the role of DCE MRI in differential diagnosis of microcalcification region remains unclear.

The purpose of our study was to prospectively evaluate the diagnostic performance of DCE MRI in the differentiation of malignant lesions from benign ones in patients with BI-RADS 3–4 microcalcifications detected by mammography.

## Materials and Methods

### Study design

This prospective study was approved by the institutional ethics committee of the First Affiliated Hospital of Nanjing Medical University. Written informed consent was given by the patients for their information to be used for our research. Furthermore, our study was also in compliance with the Helsinki Declaration.

The patients with BI-RADS 3–4 microcalcifications detected by mammography were asked to undergo DCE MR imaging of breast. The time interval of Mammography and DCE MR imaging was no more than 3 months. All mammograms were interpreted independently by two radiologists and agreement was reached after discussion. All MR images were interpreted independently by the same two readers and agreement was reached after discussion. The interobserver agreement was calculated by generalized kappa tests. All patients received open biopsy or stereotactic vacuum-assisted biopsy (SVAB). All lesions were verified by histology. The time period between MRI and histological verification of lesions was no more than one week. The positive predictive value (PPV) and negative predictive value (NPV) of mammography and breast MRI in the area of microcalcification were calculated respectively. Receiver operating characteristic (ROC) curves were calculated for mammography and breast MRI final assessment.

### Patient population

A total of 93 women (age range, 26–65 years; mean age 45.4 years) from one site between June 2010 and July 2013 were asked to participate in the study.

### Mammography protocol and interpretation

The conventional two-view mammograms (including craniocaudal and mediolateral oblique views) were obtained by clinical full-field digital mammography unit, which used molybdenum or rhodium for target and filter (Selenia, Hologic, USA). The system's autofilter mode automatically determined peak voltage and milliampere-seconds setting for the study. Microcalcifications were classified according to BI-RADS for mammographic features including calcification morphology and distribution. Images were interpreted independently by two radiologists with more than 5 years of experience in mammography. In cases of discordance, agreement was reached after discussion.

### Breast MRI protocol and interpretation

Magnetic resonance imaging was performed with patients in the prone position. The scanner was a 3.0 T system (MAGNETOM Trio, Siemens, Germany) with an 8 channel bilateral breast coil. The DCE MRI was acquired with a 3D transverse fast low angle shot T1-weighted sequence(TR/TE,4.23/1.57; matrix 448×296;slice thickness 0.9 mm; pixel resolution 1.1×0.8×0.9 mm^3^) with fat suppression. For dynamic study we imaged one pre- and five post-contrast sequences. Contrast media (Gadolinium-DTPA, BayerSchering, Germany) was administered immediately after the end of first (pre-contrast) sequence as a bolus intravenous injection at a dose of 0.1 mmol/kg and at the rate of 3 ml/s, which was followed by a 20 ml saline solution. The total time of the dynamic study was 6 minutes and 26 seconds with a temporal resolution of 1 minute. The necessary part of the examination was post-process by creating subtraction images of each post-contrast sequence and by creating maximum intensity projection (MIP) images.

Due to dynamic contrast-enhanced MR images were 3D images, while mammograms were overlapping 2D images, we selected homologous regions of MIP image compared with microcalcification regions of mammogram. MRI morphology of enhancement modality and kinetic curves were evaluated for all enhanced microcalcification regions. BI-RADS category was evaluated as final assessment for all enhanced microcalcification regions. Images were interpreted independently by two radiologists with more than 1500 interpretations experience in breast MRI. In cases of discordance, agreement was reached after discussion.

### SVAB or open surgery protocol

SVAB with 11-gauge probe (Mammotome, USA) was performed to 2 patients guided by X-ray mammography (MultiCare Platinum, Hologic, USA). The number of samples taken during the biopsy was 12 and 15. Open biopsy was performed to 91 patients by two experienced surgeons. Specimen radiography was performed routinely on all samples. The histological findings were correlated with the mammographic findings of microcalcification by radiologist and pathologist.

### Histological diagnosis

All samples were embedded in paraffin and serially cut for histological examination. Histological diagnosis was determined by the pathologist with more than ten years of experience in breast histology. Atypical ductal hyperplasia (ADH) and sclerosing adenosis were considered as benign.

### Statistical analysis

All statistical analyses were performed using software SPSS (version 11.5). An assessment of BI-RADS 4 or 5 was considered as a positive finding, while an assessment of 1, 2, or 3 was considered as negative.

The degree of interobserver agreement for mammography and breast MRI assessment between two observers was calculated by generalized kappa tests.

The PPV and NPV of the mammography and breast MRI in the area of microcalcifications were calculated respectively.

Receiver operating characteristic (ROC) curve was calculated for mammography and breast MRI final assessment respectively, and the area under the curve (AUC) was based on Hanley and McNeil method.

A *p* value less than 0.05 was considered statistically significant.

## Results

**Figure 1:**
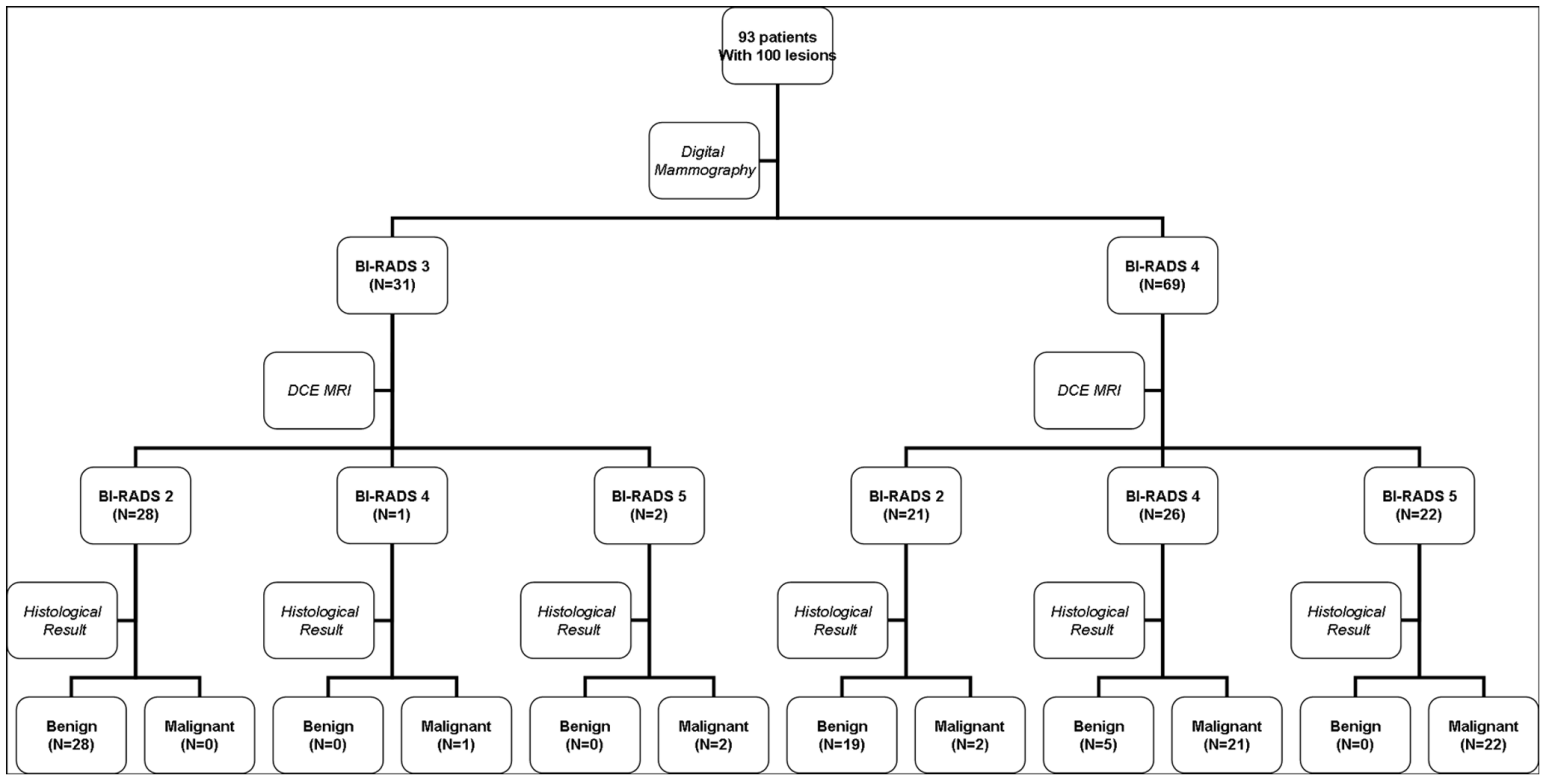
Study flowchart. BI-RADS:Breast Imaging Reporting and Data System, DCE MRI:Dynamic Contrast-Enhanced MRI.

All results were shown on the study flowchart (Figure.1).

### Histological findings

Of 2 SVAB cases, one lesion was diagnosed as ductal hyperplasia and the other was adenosis.

Of 91 open surgery cases, there were 50 lesions of benign ones, including 7 lesion of ADH, 5 lesions of sclerosing adenosis and 38 of other benign lesions, while there were 48 lesions of malignant ones, including 15 lesions of DCIS, 9 lesions of invasive ductal carcinoma with DCIS component, 2 lesions of invasive ductal carcinoma combined with mucinous adenocarcinoma and 22 lesions of invasive ductal carcinoma (Table 1).

**Table 1:** Distribution of histological findings in 100 lesions.

Histological types	Grade I	Grade II	Grade III	Total
Malignant				48
IDC	1	15	6	22
IDC+MAC		2	-	2
IDC+DCIS	-	5	4	9
DCIS	4	3	8	15
Benign				52
ADH	-	-	-	7
Sclerosing adenosis	-	-	-	5
Other benign disorder	-	-	-	40
Total				100

IDC invasive ductal carcinoma, MAC mucinous adenocarcinoma, DCIS ductal carcinoma in situ, ADH atypical ductal hyperplasia. Dash(-) indicates none.

### Mammographic findings

There were 100 lesions, including 31 BI-RADS 3 (31.0%) and 69 BI-RADS 4 (69.0%). The interobserver agreement of mammography diagnostic performance was good (k = 0.862, *p*<0.05). There were 86 lesions of pure microcalcification, 12 lesions of mass with microcalcification, 1 lesion of spiculation with microcalcification and 1 lesion of architecture distortion with microcalcification (Table 2). The PPV and NPV of mammography is 65.2% (45/69) and 90.3% (28/31).

**Table 2:** BI-RADS category for mammographic microcalcification features.

Features	BI-RADS category				
	3		4		Total
	Benign	Malignant	Benign	Malignant	
Pure microcalcification	25(29.1)	3(3.5)	24(27.9)	34(39.5)	86
Mass with microcalcification	2(16.7)	-	-	10(83.3)	12
Spiculation with microcalcification	1(100.0)	-	-	-	1
Architectural distortion with microcalcification	-	-	-	1(100.0)	1
Total	28	3	24	45	100

Numbers in parentheses are percentages of all lesions with that feature classified in the specified final assessment category. Dash(-) indicates none.

### Dynamic contrast-enhanced MR imaging findings

As to the enhancement modality of microcacification region, there were 39 lesions of focus enhancement, 34 lesions of mass-like or nodule-like enhancement, 7 lesions of ductal or linear enhancement, 15 lesions of segmental enhancement and 5 lesions of regional enhancement. As to the Time-intensity curve assessment, there were 54 lesions of persistent form (Type I), 23 lesions of plateau form (Type II) and 23 lesions of washout form (Type III)(Table 3).

**Table 3:** Characteristics of HR-DCE MR imaging enhancement.

Enhancement modality	BI-RADS category						
	2		4		5		Total
	Benign	Malignant	Benign	Malignant	Benign	Malignant	
Focus & foci	37(94.9)	2(5.1)	-	-	-	-	39
Mass & nodule	6(17.6)	-	4(11.8)	6(17.6)	-	18(53.0)	34
Ductal	-	-	1(14.3)	6(85.7)	-	-	7
Segmental	4(26.7)	-	-	8(53.3)	-	3(20.0)	15
Regional	-	-	-	2(40.0)	-	3(60.0)	5
total	47	2	5	22	-	24	100
**Time-intensity curve**							
Persistent form (Type I)	44(81.5)	2(3.7)	2(3.7)	5(9.3)	-	1(1.8)	54
Plateau form (Type II)	3(13.0)	-	3(13.0)	7(30.5)	-	10(43.5)	23
Washout form (Type III)	-	-	-	10(43.5)	-	13(56.5)	23
total	47	2	5	22	-	24	100

Numbers in parentheses are percentages of all lesions with that feature classified in the specified final assessment category. Dash(-) indicates none.

**Figure 2:**
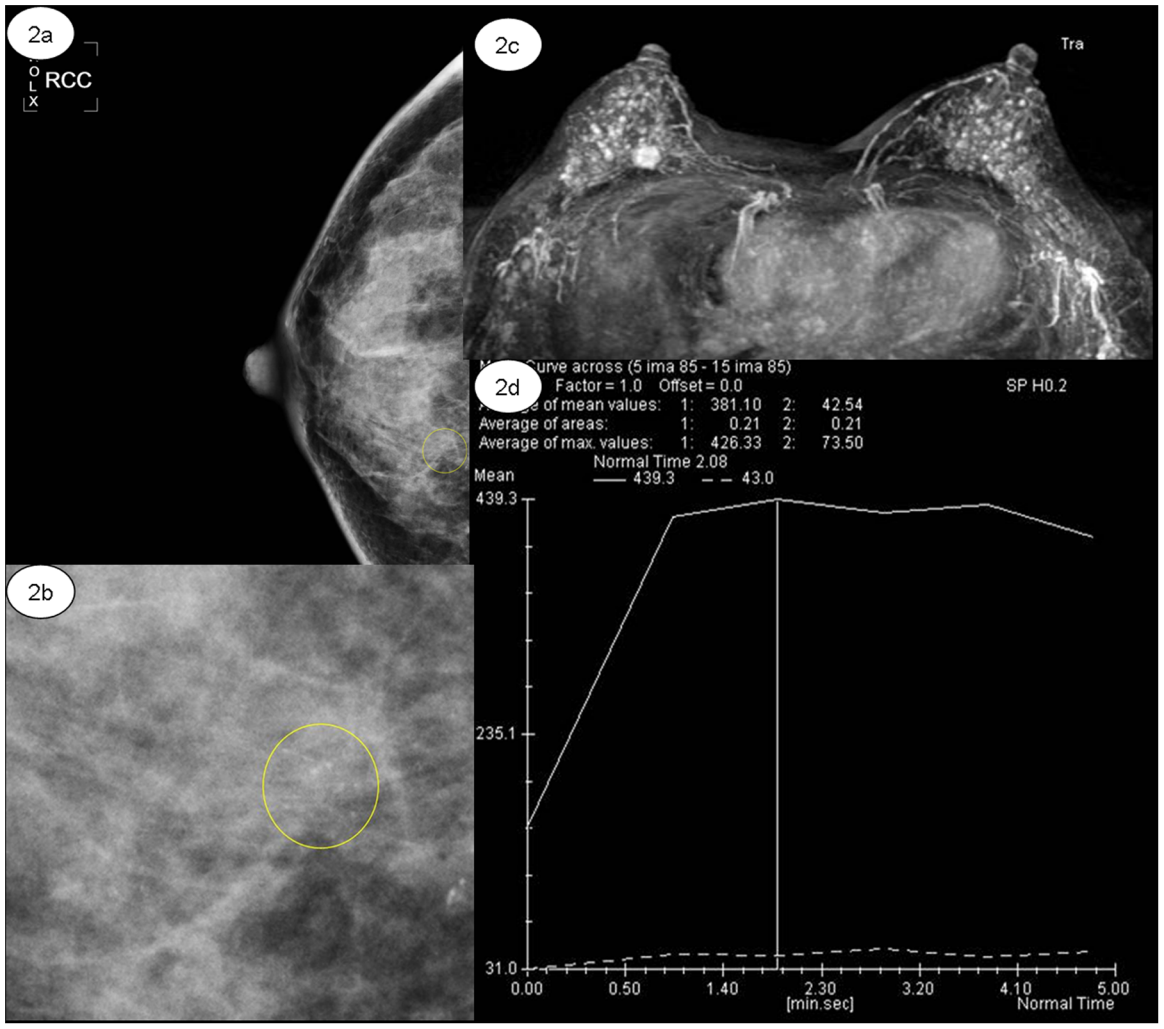
44-year-old woman with invasive ductal carcinoma in the upper inner quadrant of the right breast. (a–b) Right craniocaudal view mammogram of punctate microcalcifications with clustered distribution (yellow ring) classified as BI-RADS category 3; (c–d) Axial maximum intensity projection (MIP) of mass-like enhancement in the upper inner quadrant of right breast with plateau form of dynamic curve.

**Figure 3:**
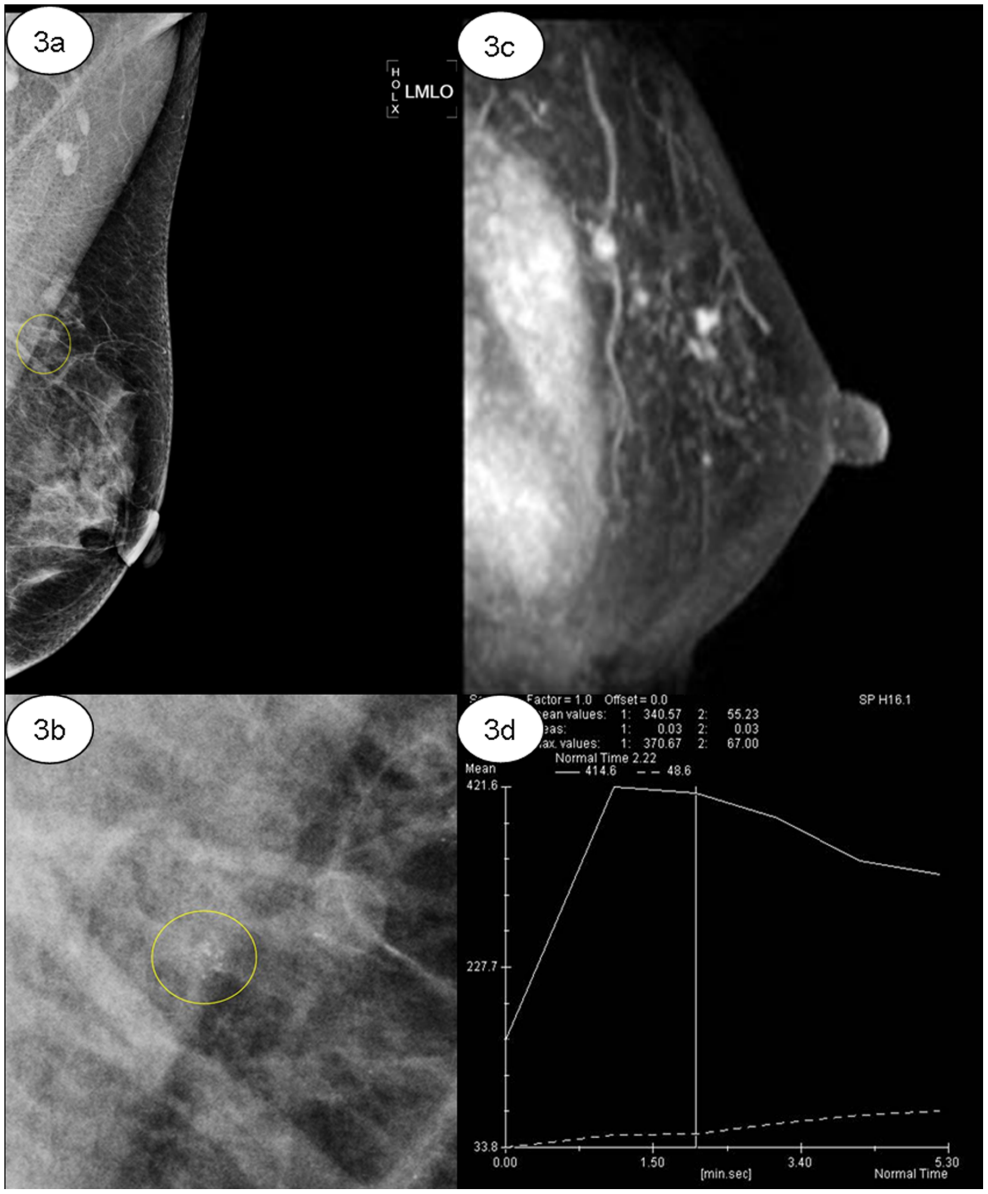
55-year-old woman with DCIS in the upper outer quadrant of the left breast. (a–b) Left media-lateral oblique view mammogram of amorphous microcalcifications with clustered distribution (yellow ring) classified as BI-RADS category 4; (c–d) Sagittal maximum intensity projection (MIP) of mass-like enhancement in the upper outer quadrant of the left breast with washout form of dynamic curve.

Among 49 lesions (49.0%, 49/100) diagnosed as BI-RADS 2 by DCE MRI, there were 47 lesions of benign and 2 lesion of malignant proven by histological findings. Among 27 lesions (27.0%, 27/100) diagnosed as BI-RADS 4, there were 22 lesions of malignant and 5 lesions of benign proven by histological findings. Among 24 lesions (24.0%, 24/100) diagnosed as BI-RADS 5, there were 24 lesions of malignant proven by histological findings. (Figure.2, 3) The interobserver agreement of breast MRI diagnostic performance was good (k = 0.900, *p*<0.05). The PPV and NPV of breast MRI was 90.2% (46/51) and 95.9% (47/49).

### Comparison of mammography and DCE MR imaging

**Figure 4:**
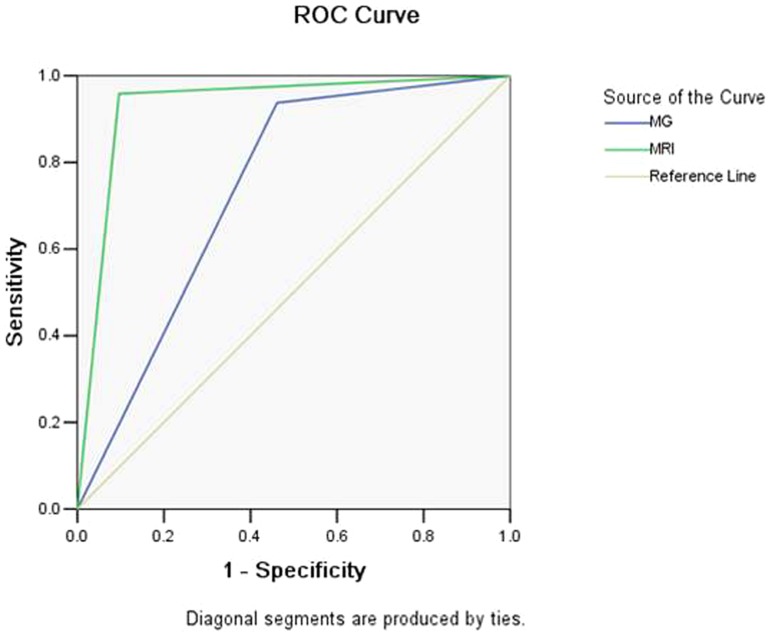
Receiver operating characteristic (ROC) curves of mammography and breast MRI.

The AUC of mammography and breast MRI assessment were 0.738 (95% CI, 0.639–0.837) and 0.931 (95% CI, 0.874–0.988) (Figure.4). The observers performed better on the breast MRI than on the mammography (*p*<0.05).

Among 31 lesions of BI-RADS 3 diagnosed by mammography, 3 lesions were upgraded to BI-RADS 4 by DCE MRI, which were proven to be malignant by histology, while the other 28 lesions were degraded to BI-RADS 2 which were proven to be benign. Therefore, PPV of DCE MRI was 100% (3/3) and NPV was 100% (28/28) in 31 BI-RADS 3 microcalcifications.

Among 69 lesions of BI-RADS 4 diagnosed by mammography, 22 lesions were upgraded to BI-RADS 5 by DCE MRI, which were verified of malignant ones; 21 lesions degraded to BI-RADS 2 by DCE MRI proven of 19 benign and 2 malignant ones and 26 lesions sustained of BI-RADS 4 verified of 21 malignant ones and 5 benign ones. Therefore, the PPV of DCE MRI was 89.6% (43/48) and NPV was 90.5% (19/21) in 69 BI-RADS 4 microcalcifications (Table 4).

**Table 4:** Comparison of BI-RADS category assessment of mammography and HR-DCE MRI.

MG	MRI						Total
	BI-RADS 2		BI-RADS4		BI-RADS 5		
	Benign	Malignant	Benign	Malignant	Benign	Malignant	
BI-RADS 3	28(90.3)	-	-	1(3.2)	-	2(6.5)	31
BI-RADS 4	19(27.5)	2(2.9)	5(7.2)	21(30.4)	-	22(32.0)	69
Total	47	2	5	22	-	24	100

Numbers in parentheses are percentages of all lesions with that feature classified in the specified final assessment category. Dash(-) indicates none.

## Discussion

Dynamic contrast-enhanced Magnetic Resonance Imaging provides tissue vascularity information. The assessment of breast MRI together with other imaging modalities mostly mammography, increases the sensitivity and specificity of modality greatly [9]. In our study, we focused on the area of suspicious malignant microcalcifications detected by mammography when MR images were evaluated. Combined with information of microcalcifications distribution, the DCE MR imaging is able to provide more accurate diagnosis especially for non-mass enhanced lesions with microcalcifications.

Some studies suggested dynamic contrast-enhanced breast MR imaging is not reliable in the differentiation of benign from malignant microcalcifications [10]–[13], while some studies indicated the dynamic contrast-enhanced breast MR imaging is able to differentiate benign from malignant disease associated with microcalcification with considerably greater accuracy than mammography or ultrasound [14]. This difference may come from the different population, the variation of magnetic field strength, breast coil specifications, pulse sequences and other parameters. In out study, the PPV and NPV of DCE-MRI for the BI-RADS 3 microcalcification was 100% respectively. These data showed that DCE MR imaging is sensitive to differentiate malignant lesions from BI-RADS 3 microcalcifications by the assessment of lesions' neovascularity.

The recommended rate of malignancy for BI-RADS category 3 microcalcifications is only 2% or fewer, which is based on follow-up mammograms not proven by histology results. Very little information on the result of SAVB is available for BI-RADS 3. In a study of 100 pure microcalcifications, malignancy was proved to be in 7% of BI-RADS 3 microcalcifications reported by Uematsu et al. [15]. In our study, 3 (9.7%) of 31 BI-RADS 3 microcalcifications were malignant proven by histological results. The rate was similar to the rate reported by Uematsu et al.

Previous studies reported the rate of malignancy ranged from 20 to 34% for BI-RADS 4 lesions (not only microcalcification lesions) [3], [5]–[6]. In a study of 100 pure microcalcifications, malignancy was proved to be in 48% of BI-RADS 4 microcalcifications reported by Uematsu et al. [15]. In our study, the rate of malignancy for BI-RADS 4 microcalcifications was 65.2% (45/69). The difference may come from the different populations of these studies which have different proportions of BI-RADS 4 subtypes (BI-RADS 4a, 4b and 4c).

In our study, the PPV of DCE MR imaging for BI-RADS 4 microcalcifications was 89.6% (43/48). The result suggests that DCE MR imaging is able to reduce the number of SVAB or open biopsy for the BI-RADS 4 microcalcifications.

As to the contrast-enhanced breast MRI, there is strong evidence that the sensitivity is greater than the specificity of other techniques of breast imaging [16]. There is still a source of great controversy as to the specificity of breast MRI [17]–[21].

In our study, five benign microcalcification lesions (2 lesions of adenosis, one lesion of ADH, one lesion of sclerosing adenosis and one lesion of intraductal papilloma) were interpreted as positive on MR imaging. Four lesions were mass-like enhancement, including three with plateau type of time-intensity curve and one with persistent type of time-intensity curve. It's probably true that the margin and contrast agent kinetics of mass lesions is helpful for diagnosis. But in clinical practice, it is not always true because some complex adenosis looks like irregular mass and the plateau form of kinetic curve exists not only in malignant lesions but also in benign ones.

Baltzer et al [22] reported non-mass lesions were identified as a challenging subgroup causing a high proportion of false-positive diagnoses at breast MRI. They identified 48% false-positive findings among all non-mass lesions. Tozaki et al [23] also found a high fraction of false-positive findings among 30 non-mass lesions (40%, 12 of 30). And in our study, most of pure DCIS (93.3%, 14/15) were enhanced with non-mass modality, including 1 with spot enhancement, 5 with segmental enhancement, 5 with ductal enhancement and 3 with regional enhancement. However, our study showed only 1.5% (1/66) false-positive findings. So we think high spatial resolution of DCE MRI with thinner slice thickness may be helpful for differential diagnosis of non-mass lesions.

In our study, one malignant microcalcification lesion of high-grade DCIS (grade 2–3, less than 1 mm) was interpreted as negative on MR imaging. In this false-negative case, diffuse parenchymal enhancement of both breasts (symmetric enhancement) was noted. This sign is similar to previous studies' [24]–[26]. Small tumor size and diffuse parenchymal enhancement lower the specificity of breast MR imaging. In premenopausal women, performing breast MR imaging within the second week of the menstrual cycle may improve the sensitivity and specificity of dynamic contrast-enhanced breast MR imaging because diffuse enhancement were reduced [24], [27].

Recently, a high-resolution dedicated breast PET [MAMmography with Molecular Imaging (MAMMI)] has been developed to improve primary tumor detection and characterization [28]. Some studies reported comparison between breast PET, conventional PET/CT and MRI with good results [29], [30]. The dedicated breast PET may be useful to differentiate malignant lesions from benign ones in patients with BI-RADS 3–4 microcalcifications.

Several limitations still existed in this study. First, the sample size was small, especially for the cases with BI-RADS 3 microcalcifications. The high rate of malignancy in BI-RADS 3 microcalcifications may be caused by the small sample size. Therefore, future studies with bigger sample size are still needed to confirm our findings. Second, breast MRI was performed without being blinded to mammographic findings. This artificially inflates the diagnostic performance of breast MRI.

## Conclusions

During the clinical treatment of BI-RADS 3 and 4 microcalcifications, DCE MR imaging of breast is able to be applied to predict the risk of malignance before follow-up for BI-RADS 3 and biopsy for BI-RADS 4. DCE MR imaging of breast is an effective and non-invasive method in diagnosis. In addition, we should be careful to assess suspiciously malignant microcalcification region (BI-RADS 4) with the background of diffused enhancement modality in DCE MRI.
